# Socio-economic conditions affect health-related quality of life, during recovery from acute SARS-CoV-2 infection: Results from the VASCO study (VAriabili Socioeconomiche e COVID-19), on the “Surviving-COVID” cohort, from Bergamo (Italy)

**DOI:** 10.1186/s12879-024-09502-x

**Published:** 2024-08-12

**Authors:** Simone Vasilij Benatti, Serena Venturelli, Roberto Buzzetti, Francesca Binda, Luca Belotti, Laura Soavi, Ave Maria Biffi, Maria Simonetta Spada, Monica Casati, Marco Rizzi, on behalf of the SURVIVING COVID study group, Maria Grazia Alessio, Chiara Ambaglio, Tiziano Barbui, Pietro Andrea Bonaffini, Emi Bondi, Giorgia Camera, Greta Carioli, Alessandra Carobbio, Simonetta Cesa, Caterina Conti, Roberto Cosentini, Giacomo Crotti, Anna Falanga, Simonetta Gerevini, Arianna Ghirardi, Andrea Giammarresi, Giuseppe Greco, Gianluca Imeri, Marina Marchetti, Claudia Marinaro, Aicha Ouabou, Ramona Pellegrini, Giulia Previtali, Giampaolo Quinzan, Alessandro Rossini, Michela Seghezzi, Maria Sessa, Roberta Severgnini, Claudia Suardi, Adriana Zanoletti, Alberto Zucchi, Gianluca Zuglian

**Affiliations:** 1 Infectious Diseases Service, ASST “Papa Giovanni XXIII”, Bergamo, Italy; 2 Clinic of Infectious Diseases, Dept of Health Sciences, University of Milan, ASST Santi Paolo e Carlo, Milan, Italy; 3 School of MedicineUniversity of Milano Bicocca, Milano, Italy; 4 Clinical Psychology Service, ASST “Papa Giovanni XXIII”, Bergamo, Italy; 5 Clinical Research Unit for Healthcare Professions, ASST “Papa Giovanni XXIII”, Bergamo, Italy; 6 Freelance epidemiologist, Bergamo, Italy

**Keywords:** SARS-CoV-2, COVID-19, Epidemiology, Healthcare disparities, Socioeconomic status, Health-related-quality-of-life, Mental health, SF-36, IES-R, DLCO

## Abstract

**Background:**

Recovery from acute COVID-19 may be slow and incomplete: cases of Post-Acute Sequelae of COVID (PASC) are counted in millions, worldwide. We aimed to explore if and how the pre-existing Socio-economic-status (SES) influences such recovery.

**Methods:**

We analyzed a database of 1536 consecutive patients from the first wave of COVID-19 in Italy (February-September 2020), previously admitted to our referral hospital, and followed-up in a dedicated multidisciplinary intervention. We excluded those seen earlier than 12 weeks (the conventional limit for a possible PASC syndrome), and those reporting a serious complication from the acute phase (possibly accounting for symptoms persistence). We studied whether the exposition to disadvantaged SES (estimated through the Italian Institute of Statistics’s model – ISTAT 2017) was affecting recovery outcomes, that is: symptoms (composite endpoint, i.e. at least one among: dyspnea, fatigue, myalgia, chest pain or palpitations); Health-Related-Quality-of-Life (HRQoL, as by SF-36 scale); post-traumatic-stress-disorder (as by IES-R scale); and lung structural damage (as by impaired CO diffusion, DLCO).

**Results:**

Eight-hundred and twenty-five patients were included in the analysis (median age 59 years; IQR: 50–69 years, 60.2% men), of which 499 (60.5%) were previously admitted to hospital and 27 (3.3%) to Intensive-Care Unit (ICU). Those still complaining of symptoms at follow-up were 337 (40.9%; 95%CI 37.5–42.2%), and 256 had a possible Post-Traumatic Stress Disorder (PTSD) (31%, 95%CI 28.7–35.1%). DLCO was reduced in 147 (19.6%, 95%CI 17.0–22.7%). In a multivariable model, disadvantaged SES was associated with a lower HRQoL, especially for items exploring physical health (*Limitations in physical activities*: OR = 0.65; 95%CI = 0.47 to 0.89; *p* = 0.008; AUC = 0.74) and *Bodily pain* (OR = 0.57; 95%CI = 0.40 to 0.82; *p* = 0.002; AUC = 0.74). We did not observe any association between SES and the other outcomes.

**Conclusions:**

Recovery after COVID-19 appears to be independently affected by a pre-existent socio-economic disadvantage, and clinical assessment should incorporate SES and HRQoL measurements, along with symptoms. The socioeconomic determinants of SARS-CoV-2 disease are not exclusive of the acute infection: this finding deserves further research and specific interventions.

**Supplementary Information:**

The online version contains supplementary material available at 10.1186/s12879-024-09502-x.

## Background

Individual health and life expectancy appear to be strongly influenced by socio-economic level, even in the industrialized world [1, 2]. Reports from various countries, widely differing in healthcare expenditure and accessibility, highlight a linkage between COVID-19-related mortality and socio-economic disadvantage [3–7].

A substantial number of survivors to SARS-CoV-2 infection suffer from “Post-acute Sequelae of COVID-19” (PASC): a group of poorly understood clinical conditions [8, 9], ranging from mild to severely debilitating, and possibly afflicting thousands of patients, even after more than one year since the acute infection [10–12]. Its incidence across different countries is uncharted, also due to the lack of strict diagnostic criteria [13–15]: in fact, the official definition of PASC (first formulated only in December 2020 by NICE [16] and substantially endorsed by WHO, employing a Delphi Consensus initiative in October 2021 [17], is exclusively relying on self-reported symptoms, and no diagnostic biomarker or test is available [18].

It seems very reasonable that a more advantaged socioeconomic status (SES) could positively influence speed and completeness of recovery, after COVID-19, and evidence is increasing about that [19–25].

Our aim was to explore how SES interacted with recovery, on a very special population we had the unique chance to study in-person, after hospital discharge from our institution (“Papa Giovanni XXIII” Hospital, in Bergamo, Italy), in the months of the very first wave of COVID-19 epidemic outside China, right after Wuhan (the “Surviving COVID” cohort).

## Methods

### Study type

Retrospective exploratory cohort study, with single time-point multimodal assessment, of a population of adult patients recovering from COVID-19.

### Population

“Surviving COVID” was a public-funded intervention of follow-up, for the survivors to the first epidemic wave, held from 5 May 2020 to the end of November 2020, at “Papa Giovanni XXIII” Hospital, the principal public hospital of the Bergamo province, Italy. A detailed description of the intervention has been already reported elsewhere (see also Supplementary Material) [26]. Briefly, we offered participation to all consecutive adult patients, admitted to the wards of the hospital or discharged (without admission) from the emergency department (ED), between February and September 2020, with a history of SARS-CoV-2 infection, confirmed by a molecular test. The aim of the intervention (one of the first for survivors to COVID-19) was, above all, to provide medical and psychological assistance, and – secondarily – to offer a multidimensional characterization of the recovery process: it consisted in a psychological interview, instrumental tests, full blood analyses, and a medical encounter. Patients were seen only once (on a two-consecutive day schedule to accommodate all the investigations and visits); the time distance from COVID-19 onset was variable, according to the availability of each patient. The “Surviving COVID” intervention allowed to collect a rich database, on which we retrospectively tested our hypothesis about SES, in the current work (VASCO analysis).

### Exposure

Pre-existing Socio-economic Status (SES), as estimated through a 9-class socio-economic model, developed by Italian National Institute of Statistics (ISTAT) [27]. The information required by the model is: nationality (Italian *versus* other), level of education (up to secondary school *versus* high school *versus* university or above), number of members in the household, and occupation. Such information was obtained from each patient through a specific Socio-economic Questionnaire (SQ) to be filled in. For each patient, we also collected information about the occurrence of other COVID-19 cases, requiring hospital admission, in the same household.

### Outcomes

The quality and amount of recovery from COVID-19. We define “recovery from acute COVID-19”, as the dynamic process of returning to the pre-COVID conditions, after conclusion of the acute phase of the infection. For practical issues, we complied to the definition by WHO [17], considering 12 weeks since onset as the maximum time for a physiologic recovery process, beneath which – in case of persisting symptoms - a PASC condition is to be considered. Since such process of recovery appears variable in quality and dimensions, among individuals, and there’s no single marker certifying its accomplishment, we attempted its evaluation, through the following variables:

- Presence and type of symptoms on the day of the medical encounter (primary outcome): for the current analysis purposes, we created a composite endpoint called “Physical symptoms”, positive in case of at least one among: fatigue, dyspnoea, chest pain, myalgia and palpitations.
- Diffuse Capacity of the Lungs for Carbon monoxide (DLCO) at follow-up: abnormal if lower than 80% of the expected value (corrected for sex, age, height and ethnicity);
- IES-R scale [28], as assessed by a psychologist trained in this, on a dedicated encounter: a score higher than 33 was considered suggestive of a post-traumatic stress disorder (PTSD);
- SF-36 scale [29, 30], with its 8 sub-questionnaires, considered pathologic if resulting 0 or 1.
- Brief Fatigue Inventory (BFI) [31] and Barthel index [32], scored by patients for their current and pre-COVID condition, and categorized into deteriorated or unchanged.

Maximal O2-need during the acute phase, hospital admission, and ICU admission were taken as proxies for acute-phase clinical severity. Maximal O2 requirement attained was categorized as: A = no O2 need, B = nasal prongs, C = mask with high flow, but no Positive End-Expiratory Pressure (PEEP), D = all other cases, namely: Continuous Positive Air Pressure (CPAP), High Flow Nasal Cannula (HFNC), Non-Invasive Mechanical Ventilation (NIMV), mechanical ventilation (MV) or Extracorporeal Membrane Oxygenation (ECMO). We grouped together under “D” such different support modalities, because during the first epidemic wave our hospital system was so dramatically overcharged, that many patients, who under other circumstances would have qualified for ICU admission, were eventually treated by CPAP, HFNC or NIMV in ordinary wards.

Any serious complication occurring during the acute phase of the SARS-Co-2 infection was recorded and categorized as follows:

- neurologic (e.g. stroke, encephalitis, Guillain − Barré syndrome, polyneuropathy).
- cardiac (e.g. arrhythmia, ischemia, myocarditis).
- pulmonary (e.g. bacterial pneumonia, pleural effusion, pneumothorax).
- thrombotic (e.g. pulmonary embolism, deep-vein/arterial thrombosis).
- infectious (e.g. COVID-associated pulmonary aspergillosis, hospital acquired infections…).

Data were collected in a Microsoft Access database.

### Statistical analysis

A descriptive analysis was performed for each variable. For continuous ones, the mean, the standard deviation, the minimum and maximum values, the median and quartiles are provided; for categorical ones we reported the distribution of frequencies. For simplicity, in the inferential analysis we transformed all continuous variables into categorical ones. In particular, for age and time from onset we took as a cut-off the median of the distribution, after excluding multimodality (above or below 60 years of age; time from onset to follow-up above or below 133 days). For BMI and DLCO reduction we adopted commonly employed cut-offs (for overweight a BMI > = 30 Kg/m^2^, for lung interstitial damage a reduction in DLCO > = 80% of expected value). Similarly, the 9-classes SES categorization was simplified in some cases, clustering data into three income brackets (low income - middle income - high income), as suggested by ISTAT itself (Table 1 in the Supplementary Material), and the three brackets treated as a continuous variable with 3 levels.

A univariable exploratory analysis verified the association between each of the predictive variables with each of the outcome ones. The appropriate statistical tests were performed (Chi-square test or Fisher’s exact test for tables of categorical variables; Student’s *t* test for comparison between means). The association between SES (social class from 1 to 9, or –for some analysis - income bracket: low-middle-high) and outcomes was estimated by the Odds Ratio, with its 95% confidence interval.

Due to the high number of exploratory hypothesis (involving 8 independent groups of baseline variables and 2 independent groups of outcomes – please see Supplementary Material for further details), the significance level was set at 0.003, according to Bonferroni. Despite such a significance cut-off, the Chi-square power for an absolute risk difference between groups of 0.15 (15%) remains above 93%.

We created various logistic analysis models, including as independent variables those found significant in the univariable analysis and/or most reasonably involved in determining the outcomes. As our follow-up intervention was not at time-fixed intervals (while obviously the recovery process is time-dependent), we also included in the model time to follow-up.

The goodness of the fit was estimated through the Hosmer-Lemeshow tests, and the diagnostic ability through the Area Under The Receiver Operating Characteristics (AUROC).

Records with missing values were excluded from the corresponding analyses. Continuous variables were included in the logistic models, assuming a linear relationship, after graphical checking of their distribution.

All analysis were done on Excel and on his XLStat package extension.

## Results

From 22nd February 2020 to 30th September 2020, 3,052 patients with SARS-CoV-2 infection sought care at our hospital, but just 1,536 of the 2,391 survivors were finally enrolled in the “Surviving COVID” database, because of refusal or loss of contact.

For the current post-hoc analysis, we excluded patients (*n* = 201) followed-up earlier than 12 weeks from onset, considering that this was a reasonable time limit for the recovery process to be still not complete (as suggested also in the PASC definition by NICE) [16]. Our aim was to focus on the recovery from SARS-CoV-2 infection, so we also excluded patients having experienced any serious complication during the acute phase, and possibly explaining their symptoms: in fact, such complication might have arisen, due to multiple and various reasons, even not directly attributable to SARS-CoV-2 infection.

**Fig. 1:**
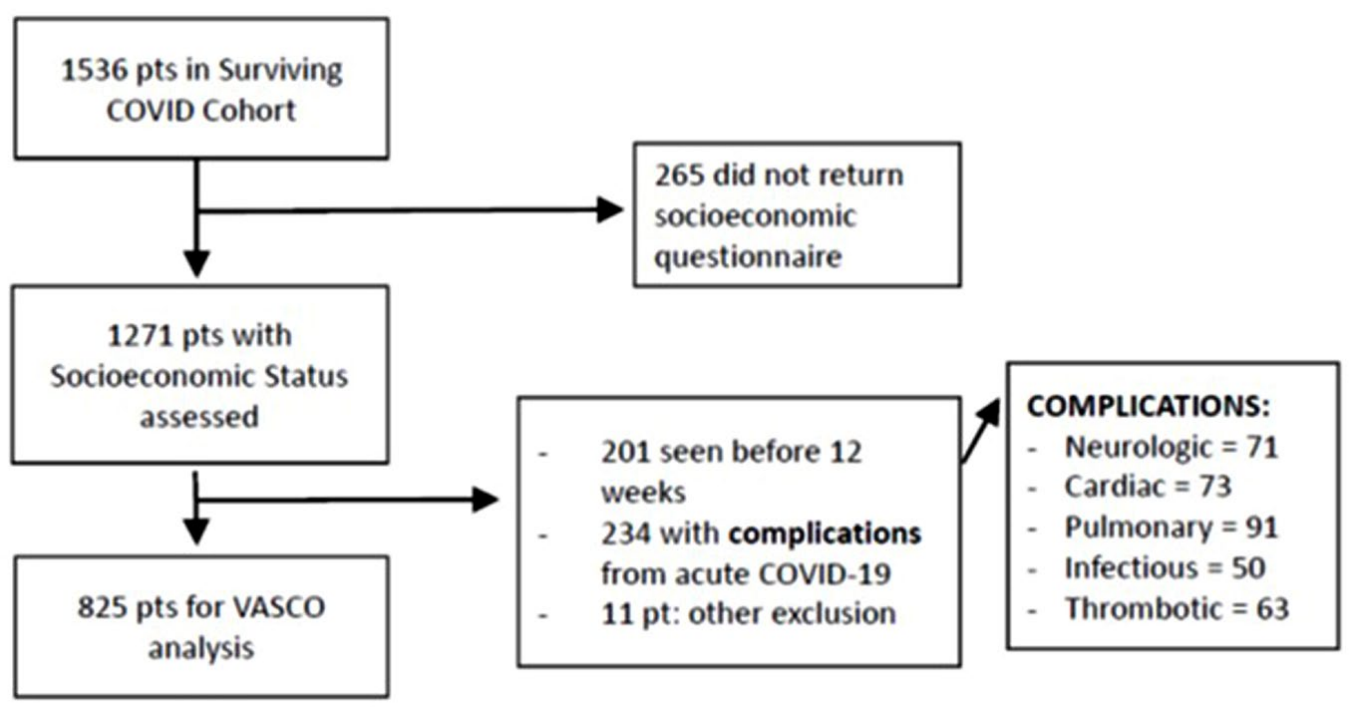
Attrition Cascade Diagram

We also excluded: five acutely asymptomatic patients (for impossibility in establishing onset), six patients living in a nursing home, and 265 patients not returning the SQ. Finally, 825 patients were included (see also Fig. 1).

Being confronted with such a relevant attrition cascade, we looked for potential selection bias, by comparing the acute-phase maximal-O2-need, sex, age and number of comorbidities, between the 825 patients included, and those not included just because seen earlier than 12 weeks (SQ not provided by 265 of them). We did not find relevant clinical differences (results not shown). Similarly, we compared SES (income bracket) between the 825 patients included and the 446 excluded for reasons other than SQ absence. This latter comparison (not shown) allows us to say that in the final sample the three wealthier social classes were overrepresented (42% of higher income bracket vs. 34% in the excluded, *p* = 0.014).

The baseline characteristics of the included patients are summarized in Table 1.

**Table 1:** Baseline characteristics of the “Surviving-COVID” patients, enrolled in VASCO analysis

	**Full cohort** **(n = 825)**	**Maximal O2 requirement (n = 823)**				*P*
		Room air (n = 354 = 43%)	Low flow (n = 169 = 21%)	High flow (n = 167 = 20%)	CPAP, HFNC, MV, ECMO (n = 133 = 16%)	
Age, mean (SD)	58.7 (13.6)	54.4 (13.7)	63.2 (12.3)	62.1 (12.8)	60.3 (12.7)	**< 0.001**
Sex						**< 0.001**
Females, n (%)	328 (40%)	191 (54%)	71 (42%)	31 (19%)	34 (26%)	
Males, n (%)	497 (60%)	163 (46%)	98 (58%)	136 (81%)	99 (74%)	
BMI (Kg/m2), mean (SD)	(n = 612) 26.7 (4.3)	25.4 (3.9)	26.5 (4.2)	27.3 (4.4)	28.0 (4.4)	**< 0.001**
**Cigarette smoke, n (%)**						
(current or past)	274 (33%)	114 (32%)	61 (36%)	49 (29%)	50 (38%)	0.381
Comorbidities, n (%)	(n = 823)					**< 0.001**
None	465 (57%)	251 (71%)	74 (44%)	68 (41%)	72 (54%)	
One	212 (26%)	76 (21%)	50 (30%)	53 (32%)	33 (25%)	
More than one	146 (18%)	27 (8%)	45 (27%)	46 (28%)	28 (21%)	
Comorbidities, n (%)	(n = 823)					**< 0.001**
Hypertension	265 (32%)	72 (20%)	72 (43%)	78 (47%)	43 (32%)	
Diabetes	78 (9.5%)	15 (4%)	18 (11%)	28 (17%)	17 (13%)	
Income bracket, n (%)	(n = 802)					0.009
Low income	161 (20%)	76 (22%)	30 (19%)	29 (18%)	26 (20%)	
Median income	307 (38%)	106 (30%)	71 (44%)	73 (45%)	57 (44%)	
High income	334 (42%)	167 (48%)	61 (38%)	59 (37%)	47 (36%)	
Social class, n (%)	(n = 803)					**< 0.001**
1	37 (5%)	18 (5%)	7 (4%)	5 (3%)	7 (5%)	
2	58 (7%)	33 (9%)	8 (5%)	7 (4%)	10 (8%)	
3	24 (3%)	14 (4%)	5 (3%)	4 (2%)	1 (1%)	
4	82 (10%)	40 (11%)	16 (10%)	15 (9%)	11 (8%)	
5	30 (4%)	6 (2%)	7 (4%)	11 (7%)	6 (5%)	
6	238 (30%)	71 (20%)	58 (16%)	61 (28%)	48 (37%)	
7	115 (14%)	76 (22%)	11 (7%)	14 (9%)	14 (11%)	
8	122 (15%)	48 (14%)	28 (17%)	28 (17%)	18 (14%)	
9	97 (12%)	43 (12%)	22 (14%)	17 (10%)	15 (12%)	
Educational level, n (%)	(n = 802)					**< 0.001**
Primary or less	128 (16%)	32 (9%)	43 (27%)	29 (18%)	24 (19%)	
Secondary (years 7-8-9)	237 (30%)	92 (26%)	38 (24%)	62 (38%)	45 (35%)	
Secondary (years 10 to 13)	284 (36%)	150 (43%)	47 (29%)	49 (30%)	38 (30%)	
University or above	153 (19%)	77 (22%)	33 (21%)	22 (14%)	21 (16%)	
Born abroad, n (%)	59 (7.2%)	28 (8%)	7 (4%)	13 (8%)	11 (8%)	0.398
Occupation, n (%)	(n = 809)					**< 0.001**
- unemployed	49 (6%)	28 (8%)	7 (4%)	6 (4%)	8 (6%)	
- employed	457 (56%)	232 (66%)	73 (45%)	83 (50%)	69 (53%)	
- retired	303 (37%)	89 (26%)	84 (51%)	76 (46%)	54 (41%)	

LEGEND: O2: oxygen; *SD*: standard deviation; *CPAP*: Continuous Positive Air pressure; *HFNC*: High Flow Nasal Cannula; *MV*: mechanical ventilation; *ECMO*: extra-corporeal membrane oxygenation; *BMI*: body-mass index – in bold characters the significant results

At follow-up evaluation, after a median of 133 days (IQR 115–171) from onset, 337 (40.9%, 95% CI 37.5–42.2%) of the participants complained of physical symptoms (“Symptomatic patients”, in the Tables), mainly of fatigue in 248 (30%, 95% CI 27.0–33.3%), and dyspnea in 126 (15.3%, 95% CI 13.0–17.9%). DLCO (*n* = 748) was less than 80% of the expected in 147 (19.7%, 95% CI 17.0–22.7%). For DLCO, we observed 77 (9%) missing values, principally because of insufficient collaboration in the test, due to old age and disability.

A loss of autonomy (reduction of Barthel score, *n* = 820) occurred in 46 patients (5.6%, 95% CI 4.2–7.4%), whereas an increase in fatigue (BFI score, *n* = 820) in 415 (50.6%, 95% CI 47.2–54.0%): this was the most prevalent pathological outcome.

IES-R (*n* = 805 patients) identified 256 patients (31.8%, 95% CI 28.7–35.1%), with a post-traumatic stress condition.

SF-36 items (*n* = 800) gave the following pathologic results (by decreasing prevalence):

- *Limitations in usual role activities for physical health problems*: 35.3% (95% CI 32.0–38.6%);
- *Limitations in usual role activities for emotional problems*: 30.1% (95% CI 27.1–33.4%);
- *Limitations in social activities for physical or emotional problems*: 23.9% (95% CI 21.1–27.0%);
- *Limitations in physical activities for health problems*: 21.1% (95% CI 18.4–24.1%);
- *Vitality (energy and fatigue)*: 17.4% (95% CI 14.9–20.2%);
- *Bodily pain*: 17.0% (95% CI 14.6–19.8%);
- *General health perceptions*: 14.8% (95% CI 12.5–17.4%);
- *General mental health (psychological distress and well-being)*: 9.5% (95% CI 7.7–11.8%).

### Univariable analysis

**Fig. 2:**
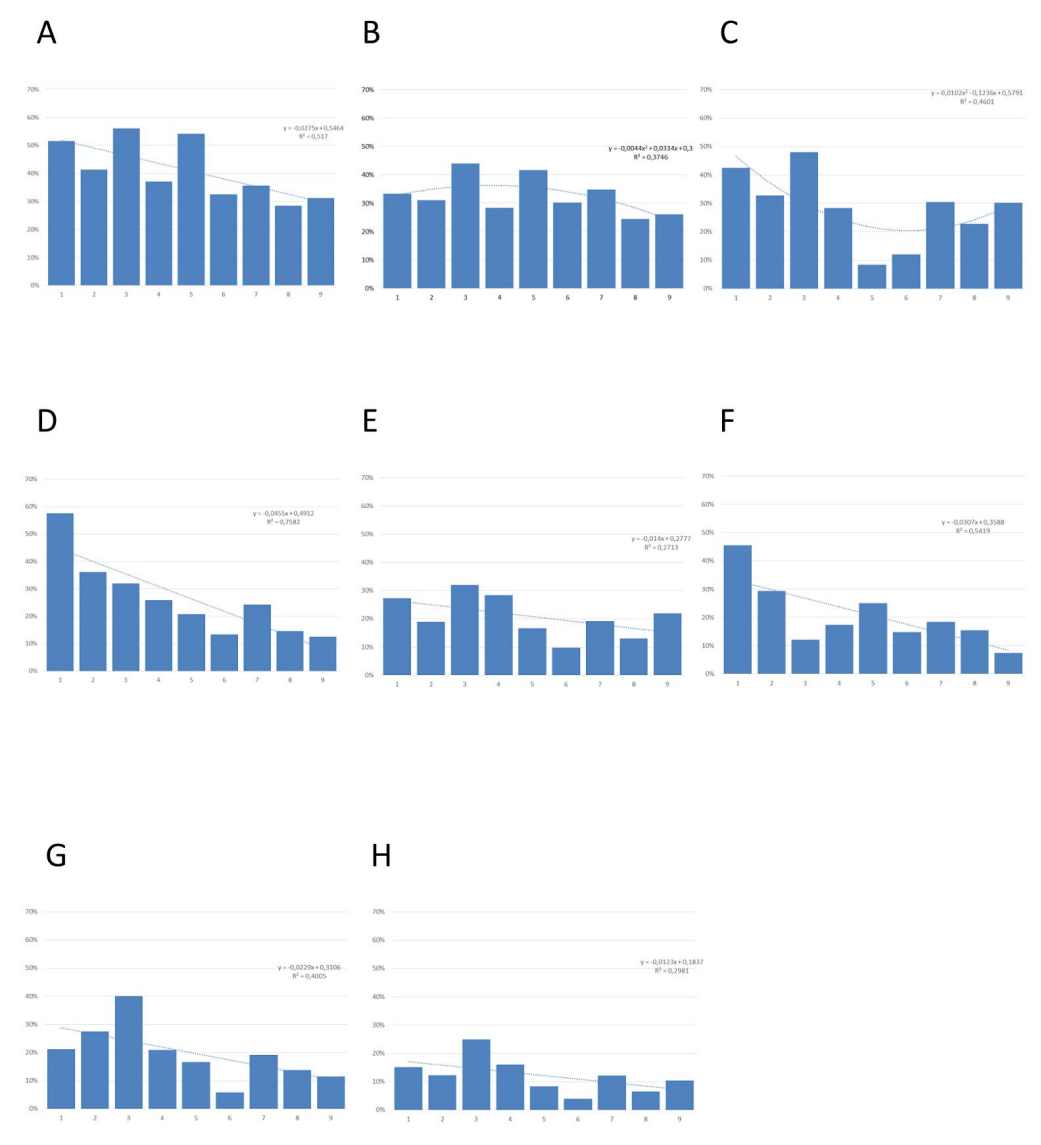
HRQoL reduction prevalence in all items of SF-36 scale, across socio-economic class from 1 to 9. Dashed line: interpolating functions (regression lines; except for B and C: second degree polynomials); R2: coefficient of determination (**A** - *Limitations in usual role activities because of physical health problems*; **B** - *Limitations in usual role activities because of emotional problems*; **C** - *Limitations in social activities because of physical or emotional problems*; **D** - *Limitations in physical activities because of health problems*; **E** - *Vitality (energy and fatigue)*; **F** - *Bodily pain*; **G** - *General health perceptions*; **H** - *General mental health*)

No one of the recovery outcomes considered was significantly associated to SES indicators, except for five items of SF-36, where a significant decrease in pathologic results was observed, passing from class 1 (most disadvantaged) to 9 (most advantaged). In particular (see also Fig. 2):

A. *Limitations in usual role activities for physical health problems*: from 51.5 to 31.3% (*X*^2^ 17.24; *p* = 0.028);
B. *Limitations in usual role activities for emotional problems*: from 33.3 to 26.0% (*X*^2^ 7.95; *p* = 0.439);
C. *Limitations in social activities for physical or emotional problems*: from 42.4%, to 30.2% (*X*^2^ 42.77; *p* < 0.001);
D. *Limitations in physical activities for health problems*: from 57.6%, to 12.5% (*X*^2^ 54.05; *p* < 0.001);
E. *Vitality (energy and fatigue)*: from 27.3%, to 21.9% (*X*^2^ 25.15; *p* = 0.001);
F. *Bodily pain*: from 45.5%, to 7.3% (*X*^2^ 33.78; *p* < 0.001);
G. *General health perceptions*: from 21.2%, to 11.5% (*X*^2^ 40.42; *p* < 0.001);
H. *General mental health (psychological distress and well-being)*: from 15.2%, to 10.4% – *X*^2^ 22.67; *p* < 0.004.

Notably, no association was found between income bracket (*X*^2^ 1.67, *p* = 0.434) and the composite endpoint “Physical symptoms”, which instead was associated with age younger than 60 (46.4%, vs. 34.7% – *X*^2^ 11.71; *p* = 0.001), female sex (50.6% in females, vs. 34.3% – *X*^2^ 21.47; *p* < 0.001), and ICU admission (63.0% in those admitted, vs. 40.1% – *X*^2^ 5.65; *p* = 0.018).

The principal univariable associations for the composite outcome “Physical Symptoms” are summarized in Table 2 in the Supplementary Material.

### Multivariable analysis

The only pathologic outcome associated with disadvantaged SES was SF-36: specifically, in two of its items, those exploring the physical domains: *Limitations in physical activities because of health problems* (OR = 0.65; 95%CI = 0.47 to 0.89; *p* = 0.008, albeit not below 0.003) and *Bodily pain* (OR = 0.57; 95%CI = 0.40 to 0.82; *p* = 0.002) – see also Tables 2 and 3). The goodness of fit of the multivariable model for those two outcomes was good, as estimated by the Hosmer-Lemeshow test: *Limitations in physical activities because of health problems* (AUC = 0.74), *Bodily pain* (AUC = 0.74). Having set a very tight threshold for significancy (alpha = 0.003), we created a second multivariable model, including a restricted number of covariates: it confirmed a strong (and very significant) association between disadvantaged SES and reduced HRQoL in the same two items of SF-36: *Limitation in physical activities because of health problems* (for higher social classes towards pathologic result): OR = 0.84, 95%CI = 0.78 to 0.91, *p* < 0.001; and *Bodily Pain*: OR = 0.86, 95%CI = 0.79 to 0.93, *p* < 0.001.

**Table 2:** Follow-up results: multivariable associations for SF-36 scale

SF-36 ITEMS	*Limitation in physical activities because of health problems*				*Limitation in usual role activities because of physical health problems*				*Bodliy pain*				*General health perception*			
	OR	95%CI		*p*	OR	95%CI		*p*	OR	95%CI		*p*	OR	95%CI		*p*
Age below 60 years	1.83	1.03	3.24	0.039	1.36	0.87	2.14	0.181	2.93	1.53	5.63	0.001	5.57	2.57	12.09	**< 0.001**
Male Sex	0.78	0.52	1.18	0.246	0.76	0.54	1.06	0.106	1.48	0.94	2.33	0.860	0.93	0.58	1.50	0.774
Born outside of Italy	1.32	0.70	2.50	0.389	1.18	0.65	2.12	0.583	1.29	0.64	2.60	0.481	0.49	0.20	1.18	0.112
BMI below 30 (Kg/m^2^)	0.63	0.39	1.02	0.061	0.78	0.53	1.16	0.228	1.16	0.67	2.01	0.592	0.89	0.48	1.64	0.710
Non smokers	0.84	0.56	1.26	0.396	1.16	0.83	1.60	0.384	0.54	0.36	0.82	0.004	0.69	0.44	1.09	0.108
Presence of comorbidities	1.52	0.91	2.51	0.107	1.37	0.94	1.99	0.104	1.47	0.87	2.47	0.148	3.57	2.07	6.15	**< 0.001**
History of diabetes	0.90	0.41	2.01	0.806	0.77	0.42	1.40	0.387	0.98	0.43	2.24	0.956	0.60	0.24	1.53	0.286
History of hypertension	0.60	0.29	1.24	0.168	0.69	0.40	1.19	0.184	0.75	0.36	1.60	0.461	0.28	0.13	0.61	**0.001**
Income bracket (Low vs. Intermediate -Intermediate vs. High)	0.65	0.47	0.89	0.008	0.88	0.66	1.19	0.408	0.57	0.40	0.82	**0.002**	0.85	0.57	1.26	0.411
Educational level, High school or above [above year 9])	0.94	0.56	1.56	0.799	0.88	0.56	1.38	0.567	1.14	0.66	1.96	0.648	1.19	0.64	2.22	0.573
Unemployment	1.68	0.69	4.12	0.255	1.18	0.54	2.55	0.682	0.84	0.31	2.26	0.727	3.79	1.30	11.07	0.015
Maximal O2 consumption	0.81	0.62	1.07	0.137	0.93	0.75	1.14	0.475	0.71	0.53	0.95	0.020	0.73	0.52	1.03	0.070
Hospital admission	0.91	0.51	1.62	0.749	0.67	0.42	1.07	0.093	0.84	0.46	1.53	0.564	1.38	0.71	2.68	0.348
ICU admission	1.30	0.45	3.78	0.629	2.43	1.04	5.70	0.040	1.39	0.44	4.38	0.576	1.93	0.55	6.80	0.304
Household member admitted for COVID	2.47	1.41	4.34	**0.002**	0.97	0.60	1.57	0.890	0.86	0.43	1.70	0.657	1.89	0.96	3.75	0.067
Time to follow-up (beyond 133 days)	1.78	1.13	2.81	0.013	1.20	0.85	1.70	0.305	2.01	1.23	3.28	0.006	0.93	0.55	1.59	0.799
AUROC	0.74				0.62				0.74				0.767			

LEGEND: OR: Odds Ratio; 95%CI: 95% Confidence Interval; BMI: Body Mass Index; ICU: Intensive Care Unit; AUROC: Area Under Receiving Operator Curve; DLCO: Diffusing Lung Capacity for Carbon Monoxide - in bold characters the significant results

**Table 3:** Follow-up results: simplified multivariable model for SF-36 scale

	SF-36: *Limitation in physical activities because of health problems*				SF-36: *Limitation in usual role activities because of physical health problems*				SF-36: *Bodily pain*				SF-36: *General health perception*			
	OR	95% CI		*p*	OR	95% CI		*p*	OR	95% CI		*p*	OR	95% CI		*p*
R²(Nagelkerke)				0.142				0.029				0.115				0.152
Hosmer-Lemeshow Statistic				0.369				0.181				0.243				0.962
Intercept				0.389				0.636				0.401				0.413
Social class (from 1 to 9)	0.839	0.777	0.907	**< 0.001**	0.927	0.866	0.991	0.026	0.857	0.788	0.932	**< 0.001**	0.906	0.831	0.988	0.026
Number of comorbidities	1.120	0.851	1.474	0.418	1.042	0.844	1.287	0.700	1.373	1.018	1.852	0.038	1.382	0.997	1.914	0.052
Male sex	0.752	0.514	1.101	0.143	0.755	0.552	1.032	0.078	1.326	0.868	2.026	0.191	0.834	0.538	1.290	0.414
Age	0.312	0.200	0.486	**< 0.001**	0.692	0.496	0.964	0.030	0.287	0.174	0.473	**< 0.001**	0.197	0.112	0.345	**< 0.001**
Number of household members admitted for COVID	1.861	1.136	3.049	0.014	0.990	0.643	1.523	0.963	0.849	0.457	1.579	0.606	1.943	1.106	3.415	0.021
Maximal O2 consumption in the acute phase (from A to D)	0.915	0.769	1.089	0.316	1.116	0.973	1.279	0.117	0.810	0.669	0.981	0.031	0.721	0.580	0.897	**0.003**
AUROC:	0.712				0.586				0.705				0.740			

LEGEND: OR: Odds Ratio; 95%CI: Confidence Interval 95%; O2: oxygen – in bold characters the significant results; AUROC: Area Under The Receiver Operating Characteristics

Other relevant multivariable associations for the outcomes are summarized in Table 4:

- “Physical symptoms” were positively associated to having at least one comorbidity (OR = 1.74; 95%CI = 1.19 to 2.55; *p* = 0.004) and to severity of the acute phase (both in terms of maximal O2 need OR = 1.32; 95%CI = 1.06 to 1.64; *p* = 0.013 - and ICU admission - OR = 2.57; 95%CI = 1.06 to 6.22; *p* = 0.036), but it was less frequent in males (OR = 0.38; 95%CI = 0.27 to 0.53; *p* < 0.001);
- “Physical symptoms” was not associated to SES (OR = 1.16; 95%CI = 0.86 to 1.56; *p* = 0.335);
- Barthel score deterioration was associated to maximal O2 need in the acute phase (OR = 1.68; 95%CI = 1.09 to 2.59; *p* = 0.018) and to an earlier time to follow-up visit (OR = 0.08; 95%CI = 0.03 to 0.22; *p* < 0.001);
- For DLCO reduction, the multivariable analysis showed an inverse association with cigarette smoke (OR = 0.66; 95%CI = 0.45 to 0.97; *p* = 0.035) and a direct one with comorbidities (OR = 1.86; 95%CI = 1.24 to 2.81; *p* = 0.003), but not with age, sex or acute phase O2 maximal need;
- For IES-R pathologic results were more favored by: female sex (OR = 0.48 for males; 95%CI = 0.34 to 0.68; *p* < 0.001), diabetes (OR = 2.25; 95%CI = 1.21 to 4.19; *p* = 0.010) and having one housemate admitted for COVID (OR = 2.25; 95%CI = 1.41 to 3.58; *p* = 0.001); interestingly, unemployment and a history of comorbidities looked protective towards PTSD (OR = 0.40 for unemployed; 95%CI = 0.18 to 0.91; *p* = 0.029 and OR = 0.65 for comorbid; 95%CI = 0.42 to 0.99; *p* = 0.045).
- Notably, no association was found among SES and acute-phase O2-need, as a proxy for severity (see Table 4)

**Table 4:** – Follow-up results: multivariable associations for Other Outcomes

Other outcomes	Decrease in Barthel Score				Pathologic IES-*R*				Presence of “Physical Symptoms”				DLCO reduction below 80% of expected				
	OR	95%CI		*p*	OR	95%CI		*p*	OR	95%CI		*p*	OR	95%CI		*p*	
Age below 60 years	0.70	0.27	1.86	0.478	1.15	0.71	1.84	0.574	1.22	0.78	1.91	0.376	0.89	0.52	1.52	0.665	
Male Sex	1.11	0.54	2.29	0.768	0.48	0.34	0.68	**< 0.001**	0.38	0.27	0.53	**< 0.001**	0.81	0.54	1.22	0.306	
Born outside of Italy	1.95	0.62	6.15	0.253	1.03	0.55	1.90	0.934	1.19	0.66	2.17	0.559	0.95	0.44	2.06	0.900	
BMI below 30 (Kg/m^2^)	1.08	0.47	2.47	0.853	1.25	0.81	1.91	0.314	0.92	0.62	1.37	0.684	1.43	0.86	2.37	0.163	
Non smokers	2.08	0.98	4.39	0.056	0.97	0.69	1.36	0.875	0.69	0.50	0.95	0.023	0.66	0.45	0.97	0.035	
Presence of comorbidities	0.96	0.46	1.99	0.904	0.65	0.42	0.99	0.045	1.74	1.19	2.55	0.004	1.86	1.24	2.81	**0.003**	
History of diabetes	0.75	0.25	2.22	0.597	2.25	1.21	4.19	0.010	0.60	0.33	1.10	0.097	0.58	0.30	1.11	0.099	
History of hypertension	1.52	0.52	4.39	0.442	1.39	0.76	2.53	0.287	0.69	0.40	1.18	0.177	0.56	0.30	1.03	0.064	
Income bracket (Low vs. Intermediate -Intermediate vs. High)	1.40	0.62	3.15	0.423	0.76	0.56	1.04	0.085	1.16	0.86	1.56	0.335	0.93	0.62	1.39	0.730	
Educational level (High school or above [above year 9])	0.58	0.18	1.84	0.353	1.03	0.65	1.64	0.897	0.69	0.44	1.09	0.111	1.04	0.59	1.84	0.888	
Unemployment	3.61	0.60	21.76	0.161	0.40	0.18	0.91	0.029	1.35	0.62	2.93	0.454	1.49	0.59	3.79	0.399	
Maximal O2 consumption	1.68	1.09	2.59	0.018	0.95	0.76	1.18	0.627	1.32	1.06	1.64	0.013	1.13	0.89	1.45	0.319	
Hospital admission	1.18	0.39	3.57	0.766	1.13	0.70	1.82	0.621	1.47	0.92	2.33	0.106	0.93	0.52	1.66	0.807	
ICU admission	1.11	0.26	4.76	0.888	0.76	0.28	2.08	0.597	2.57	1.06	6.22	0.036	1.81	0.72	4.56	0.210	
Household member admitted for COVID	2.20	0.95	5.11	0.067	2.25	1.41	3.58	**0.001**	1.32	0.82	2.14	0.249	1.33	0.77	2.30	0.300	
Time to follow-up (beyond 133 days)	0.08	0.03	0.22	**< 0.001**	0.99	0.69	1.42	0.957	1.36	0.96	1.92	0.084	0.86	0.57	1.30	0.480	
AUROC	0.84				0.67				0.68				0.68				

LEGEND: OR: Odds Ratio; 95%CI: 95% Confidence Interval; BMI: Body Mass Index; ICU: Intensive Care Unit; AUROC: Area Under Receiving Operator Curve; DLCO: Diffusing Lung Capacity forCarbonMonoxide - in bold characters the significant results

## Discussion

The COVID-19 epidemic burst in Bergamo region for the first time after China, in early 2020, and caused an unprecedented crisis of the hospital system and the society as a whole, intended to last for many months to follow [33, 34]. Our intention was to explore if pre-existing socio-economic disadvantage could shape the individual recovery.

To do so, we employed a large database from patients followed-up after receiving hospital care (not forcefully admitted, even though in the period under study our health system was in such a complete distress, for shortage of hospital resources, that a similar distinction would lose significance).

The completeness and quality of recovery, after at least 12 weeks from onset, was in-person investigated by Infectious Diseases or Internal Medicine specialists (for symptoms assessment), trained Psychologists (by means of semi quantitative scales for PTSD and HRQoL), and Physical Therapists (for Barhel scale and BFI); DLCO was measured by a Respiratory Medicine specialist.

We recorded a high prevalence of symptoms and pathologic results in BFI, Barthel’s scale, IES-R, SF-36, and DLCO measurement, which is perfectly in line with other authors’ findings [35–37].

Among these outcomes, only HRQoL resulted significantly associated with social disadvantage, specifically for its items addressing the physical dimension. Such association was independent from sex, age, BMI, number of comorbidities and time to follow-up.

Our findings support the idea that social disadvantage acted as a strong determinant of the recovery process, after acute infection by SARS-CoV-2. This could have been shaped by a reduced access to healthcare, or by its poorer quality. In addition, pre-existing or on-going behavioural and dietary factors could have played a role. For employed individuals, the impossibility of staying off from work for long periods (or the higher physical efforts required by poorer working conditions) could be in cause; or, for retired patients, a reduced access to home care and assistance. Research should be pursued in this direction.

In contrast with other studies [3–7], we could not find any association between social disadvantage and acute-phase COVID-19 severity, and this is reasonably due to the choice of excluding those patients, who experienced the most relevant acute complications.

A rich literature has flourished about PASC, but it would be inaccurate to label as “PASC” the clinical condition that we observed at follow-up, because, at the time when we started our intervention, no formal definition of PASC had already been established: for this reason, we adopted the composite endpoint of “Physical symptoms” (which, importantly, does not consider minor cognitive deficits). Anyway, even if our results are note directly transferrable to PASC conditions, it is notable that still few authors have studied how pre-existing SES is associated to PASC development, while in general it is recognized that PASC has a relevant impact on social functioning [38], working capacity [11, 39] and household finances.

Yoo and collaborators [23] investigated the effects by SES on PASC, but could not find any. In their study, though, PASC was defined through an *ad hoc* questionnaire, incorporating questions from SF-36, but not specifically targeting HRQoL. Unlike them, we examined symptoms, psychological scales and HRQoL (SF-36), each independently.

Authors from Sao Paulo, Brazil [24], found an association between symptoms at follow-up (not fulfilling the formal definition of PASC) and socio-economic deprivation.

A multicentre study on the influence of SES upon functional recovery, after ICU admission for COVID-19-related ARDS, did not find any influence by socio-economic deprivation, on respiratory functional outcomes at 6 months [25].

According to a big-data analysis by the UK Office for National Statistics, a higher prevalence of “long-COVID” (another term referring to PASC) is found in the most deprived areas of the country (as by Index of Multiple Deprivation) [21].

MacCallum-Bridges and collaborators [19], by studying a large data-base of interviewed patients in Michigan, measured a 27% higher risk of persisting symptoms, at 90 days after SARS-CoV-2 infection, in rural residents, as compared to metropolitan ones: this difference was halved after the introduction of vaccination.

Analysing a healthcare utilization database of about 214.000 Norwegian individuals aged 30 to 70, complaining of post-COVID condition (as assessed by their family doctors) within 180 days from infection, Reme et al. [20] found indications of a U-shaped association between income and the post-COVID condition (whereby individuals with middle income − 40th to 80th percentile - have higher odds for a post-COVID condition). The study explicitly excluded individuals hospitalized for COVID-19 and found a 10-times higher incidence of post-COVID in the pre-vaccine epidemic waves.

Studying an online cohort of 1,480 Californian patients, at a median of 360 days after infection, Durstenfeld [22] and colleagues could observe an increased risk of post-COVID condition in those with lower socioeconomic status/financial insecurity (OR, 1.62; 95% CI, 1.02–2.63), especially in the pre-omicron period.

Our analysis has many strengths. First of all, the population studied is unique: for the “catastrophic” nature of the events experienced, at the very beginning of the COVID pandemic and at a time when all the social inequalities produced by the pandemic had not yet firmly established. In addition, the study was entirely done in a pre-vaccine “era”, when the circulating viral variant was still the original one. The sample considered is large and well balanced among social classes, actively and systematically recruited, and directly interviewed by multidisciplinary staff, and the model we adopted, to estimate SES, is strong and validated by the main Italian Institute for demographic studies (ISTAT). Finally, the semi-quantitative measurement of HRQoL, here obtained by specifically trained professionals, accounts for a more reproducible and accurate [40] assessment than the mere symptoms list, as recognized also in other “chronic fatigue conditions” [41, 42].

We acknowledge the following limitations: a scarce representativeness of the whole population affected by SARS-CoV-2 (not admitted to hospital, nor consulting the Emergencies: our study is mainly focused on post-hospitalized patients); a significant attrition cascade; the unavailability of pre-COVID results for the scales adopted, and for DLCO.

Another minor limitation is that we did not calculate for our patients the Charlson Comorbidity Index, or other similar validated scales: that would have allowed an easier comparison with other post-COVID cohorts, in terms of comorbidities.

In particular, the high attrition cascade limited the representativeness of the final sample analysed (that shows a higher participation by the three more advantaged social classes). This notwithstanding, the final sample has a relevant share for each of the income bracket categories (Table 1), and allows us to draw reliable conclusions about SES impact on COVID-19 recovery of our patients.

Lacking a non-COVID control group, we cannot exclude that HRQoL reduction in lower classes depends on disadvantage itself, independently of the recovery from COVID. In fact, associations between HRQoL and socioeconomic disadvantage are well established, in other research settings, especially in response to acute illness (like falls in the elderly [43], or ischemic cardiac disease [44]). However, were the observed HRQoL reduction depending exclusively on socioeconomic disadvantage, one would expect it to act also on the psychological outcomes (i.e. on IES-R and on SF-36 items exploring the psychological domains), which is not apparent from our results.

A long time has passed now, since the hard times of the first COVID-19 waves all around the world, and the clinical characteristics of the disease, together with the reduced severity observed in the immunized hosts, have radically improved also the recovery process, making our results poorly transferrable to the current scenario. Nonetheless, the impact of socio-economic inequalities upon such a traumatic occurrence - as the first wave of COVID-19 has been everywhere – deserves a special attention by researchers, because similar events are far from impossible to happen again.

## Conclusions

In a detailed description of a large hospital-based cohort of post-COVID-19 patients, we observed a high prevalence of HRQoL reduction, which appears to be affected by a pre-existent socio-economic disadvantage. More studies in this direction could help to understand the mechanisms of such association. We propose that HRQoL and SES have a role in the assessment of post-COVID conditions, and that future research on PAS, or on post-COVID recovery at large, should include them.

### Electronic supplementary material

Below is the link to the electronic supplementary material.

Supplementary Material 1

## Data Availability

The datasets used and/or analysed during the current study are available from the corresponding author, on reasonable request.

## Abbreviations

ARDS: Acute Respiratory Distress Syndrome
AUROC: Area Under the Receiving Operator Curve
BMI: Body Mass Index
BFI: Brief Fatigue Inventory
COVID-19: Coronavirus Disease 2019
CPAP: Continuous Positive Airway Pressure
DLCO: Diffuse Capacity of the Lungs for Carbon monoxide
ECMO: Extra-Corporeal Membrane Oxygenation
HFNC: High Flow Nasal Cannula
HRQoL: Health-Related Quality of Life
IES-R: Impact of Event Scale - Revised
ICU: Intensive Care Unit
ISTAT: Italian Institute of Statistics
MV: Mechanical Ventilation
NICE: National Institute for Health and Care Excellence
NIMV: Non Invasive Mask Ventilation
O2: Oxygen
ONS: Office for National Statistics
OR: Odds Ratio
PASC: Post-acute sequelae of COVID
PTSD: Post-Traumatic Stress Disorder
SD: Standard Deviation
SES: Socioeconomic Status
SF-36: Short Form 36 scale
UK: United Kingdom
