# Supplementary material for: Socio-economic conditions affect health-related quality of life, during recovery from acute SARS-CoV-2 infection: Results from the VASCO study (VAriabili Socioeconomiche e COVID-19), on the “Surviving-COVID” cohort, from Bergamo (Italy)
Source: BMC Infect Dis. 2024 Aug 12;24:815. doi: 10.1186/s12879-024-09502-x (PMC11318141; doi:10.1186/s12879-024-09502-x)
Supplement: Supplementary file 1 — Supplementary Material 1 [file 12879_2024_9502_MOESM1_ESM.docx]

**SUPPLEMENTAL MATERIAL**

INDEX OF CONTENTS:

1. “SURVIVING COVID”: PATIENTS’ ENROLMENT AND CLINICAL PROCEDURES
2. Table 1: Repartition of the nine social classes defined by ISTAT (2017) into three income brackets
3. Figure 1: Cumulative curve of visits
4. Bonferroni correction for multiple testing
5. Table 2: Follow-up results: “Physical symptoms” composite outcome and univariable associations
6. Table 3: Association between SES and acute-phase severity (O2-need)

“SURVIVING COVID”: PATIENTS’ ENROLMENT AND CLINICAL PROCEDURES

Patients still in-hospital were put on a ‘waiting list’, and recalled once discharged. Enrolment was on a voluntary basis only after a double-negative nasopharyngeal swab for SARS-CoV-2 RNA, as required at that time by the Italian Health Authority. For cognitively impaired subjects, a caregiver helped in providing information about the medical history and in recalling the pre-acute episode health status.

The intervention was two-step:

**STEP 1**

Nurse-led evaluation with vital signs assessment, height and weight measurement.

Comprehensive blood tests.

Chest-X-ray (CXR), electrocardiogram (ECG), full pulmonary function testing with diffusion.

Psychological evaluation, assessment of rehabilitation needs, in an interview with a Physical Therapist. For assessment of rehabilitation needs we adopted the Barthel Index, as a measure of the individual disability [1], and the Brief Fatigue Inventory (BFI) scale, as a measure of the level of fatigue [2]. Conditions pre-existing to the acute COVID-19 episode were also scored using these two scales, by asking patients to recall their symptoms

PFT were performed according to current standards [1] by professionally trained respiratory technicians using Medical Graphics Elite Pro body box equipped with rapid gas analysers (MGC Diagnostics Corporation, USA) and interpreted by two experienced pulmonologists following current recommendations [2]. On account of COVID-19 restrictions, PFT were limited to spirometry and diffusing capacity for carbon monoxide (DLCO) [3]. Spirometric parameters comprised alveolar volume (VA), carbon monoxide transfer coefficient (KCO), forced vital capacity (FVC), forced expiratory volume in the first second (FEV1), and FEV1/FVC ratio. PFT parameters were expressed as a percentage of the predicted value (%) and considered impaired if below the lower limit of normal according to the Global Lung Function Initiative 2012 reference equations for spirometry [4] and the Global Lung Function Initiative 2017 reference equations for DLCO [5].

The psychological evaluation, administered by trained psychologists, primarily addressed the patients' response to COVID-19 and hospitalization. Its main purposes were to: 1) highlight patients' personal and interpersonal resources and 2) in case of psychological distress, refer them to specialist outpatient services considered the most suitable for their needs. Four self-report questionnaires – described in the paragraphs below – were administered, then each subject received feedback on results.

- *Impact of Events Scale-Revised (IES-R)* The authors referred to the Italian validation of the Impact of Events Scale-Revised (IES-R)[8], a 22-item self-report measure focusing on the impact of traumatic life events on health by describing 22 emotional reactions [9]. The subject is asked to indicate, on a five-point scale, how frequently each reaction has been experienced in the previous week yielding a total score between 0 and 88, with a score ≥ 33 indicating the probable presence of PTSD.
- *SF-36 Health Survey* The SF-36 Health Survey is a self-report questionnaire measuring the health-related quality of life (HR-QoL), well-known for its comprehensiveness, brevity, and high standards of reliability and validity [10,11]. It is a generic and multidimensional instrument with 36 questions that gives a measure of the impact of illnesses on several domains of the quality of life. The answers were analyzed using an algorithm developed and provided by “Mario Negri Institute”, which also published the Italian translation and validation of the questionnaire [12]. The difference between the individual score and the mean validation sample of every dimension was classified in four groups: 3 = above the mean, 2 = within 1 SD of the mean, 1 = within 2 SD of the mean, 0 = *>*2 SD of the mean. The latter was considered pathological for each domain of the scale.

**STEP 2** (three days later)

Infectious diseases consultation and, if appropriate, subsequent referral to primary care or to other specialists.

At the time when the intervention was planned, no formal definition of PASC was yet available, but patients were specifically asked about a list of symptoms, among which: fatigue, dyspnoea, chest pain, myalgia and palpitations, who later on were found as characteristic of PASC. Unfortunately, minor cognitive symptoms were not included in the list.

In addition, on Step 1 each patient received a paper form, to be filled at home and returned: this was the so-called “Socio-economical Questionnaire” (SQ), enquiring about the socio-economical characteristics of the patient (mainly: level of study, working condition, number of minors in the household). Country of birth was taken as a proxy for foreign origin. SQ collected also information about “how many people in the patient’s household had been admitted for COVID”, and “how many in the 1st degree familiars had died because of COVID”.

On step 2 a complete review of the clinical history of the acute COVID-19 episode was undertaken, and presence of the following comorbidities recorded: diabetes, hypertension, atrial fibrillation, previous myocardial infarction or revascularization, previous stroke, previous cardiac decompensation, Chronic Obstructive Pulmonary Disease (COPD), active malignancy (hematologic or solid), autoimmune disease.

Admission to hospital wards or intensive care units (ICUs) was tracked, as well as maximal O2 support attained during the admission (as a proxy for the clinical severity).

We also recorded cigarette smoking (present or past), as declared by patients, date of onset of symptoms (as remembered on the day of the follow-up visit), first date of hospital consultation with a confirmed SARS-CoV-2 infection, and date of hospital admission, if applicable.

Bonferroni correction for multiple testing

A potential pitfall of our approach, of multiple tests on the same sample, although a quite numerous one, is the problem of accidental observation of significant results. Anyway, we need to take into account that many of the adopted scales probably are note entirely independent, and this is obviously true for the SES indicators employed, as well. Following this line, we can isolate:

- 8 independent baseline variables:

- age,
- sex,
- BMI,
- smoking habit,
- comorbidities (number and type),
- SES (country of birth, education, occupation, income bracket),
- acute infection severity (time to first hospital evaluation, hospital admission, acute-phase maximal O2 consumption, duration of hospital stay, ICU admission),
- time to follow-up;

- 2 independent “outcomes-sets” (Table 2):

1. aggregating “physical symptoms”, BFI, Barthel and “DLCO reduction”;
2. aggregating the psychologic scales: SF36 and IES-R

Consequently, we corrected the alpha power for type I errors, according to Bonferroni, for the number of tested “families of hypotheses”, as follows:

corrected alpha = (0.05)/(8*2) = 0.003

Figure 1: Cumulative curve of visits


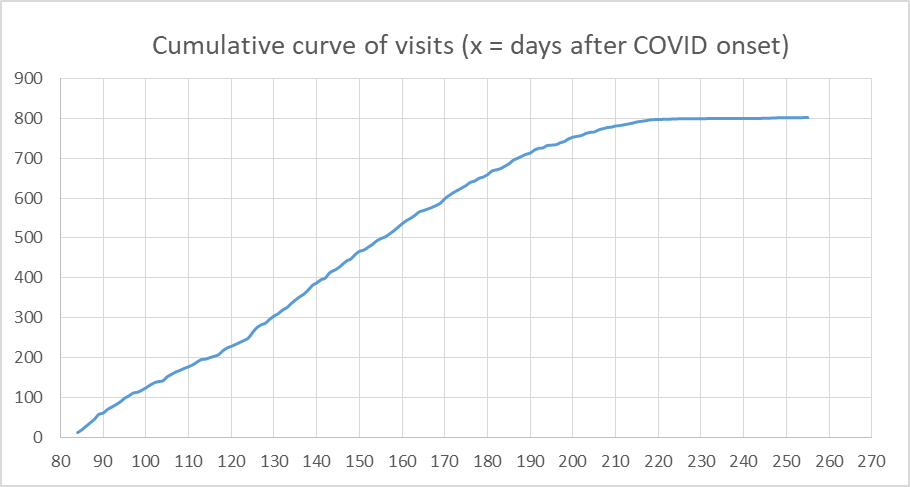


Table 1 – Repartition of the nine social classes defined by ISTAT (2017) into three income brackets

| **INCOME** | **SOCIAL CLASS** |
| --- | --- |
| LOW | 1 - low income families with foreign origin  2 - low income Italian families  3 - traditional provincial families  5 - elderly women alone and young unemployed |
| MEDIAN | 4 - young blue-collars  6 - retired workers’ families |
| HIGH | 7 - clerks’ families  8 - wealthy retirees  9 - executives |

Table 2: Follow-up results: “Physical symptoms” composite outcome and univariable associations.

|  | Non-symptomatic patients at follow-up  (n = 488 = 59%) | Symptomatic patients at follow-up  (n = 337 = 41%) | *P* |
| --- | --- | --- | --- |
| ***Baseline Characteristics*** | | | |
| Age above 60 years | 256 (52%) | 136 (40%) | ***P* = 0.001** |
| Sex  Females, n (%)  Males, n (%) | 162 (33%)  326 (67%) | 166 (49%)  171 (51%) | ***P* < 0.001** |
| Born outside of Italy, n (%) | 31 (6%) | 28 (8%) | *P* = 0.284 |
| BMI above 30 (Kg/m2) | (n = 374)  69 (18%) | (n = 236)  49 (33%) | *P* = 0.481 |
| Cigarette smoke, current or past, n (%) | 153 (31%) | 121 (36%) | *P* = 0.172 |
| Comorbidities, n (%)  None  One  More than one | 277 (57%)  129 (26%)  82 (17%) | 190 (56%)  83 (25%)  64 (19%) | *P* = 0.671 |
| Comorbidities (type), n (%)  Hypertension  Diabetes | 159 (33%)  49 (10%) | 106 (31%)  29 (9%) | *P* = 0.733  *P* = 0.488 |
| Socioeconomic class, n (%)  Low income  Median income  High income | (n = 476)  91 (19%)  190 (40%)  195 (41%) | (n = 328)  71 (22%)  117 (36%)  140 (43%) | *P* = 0.434 |
| Educational level, n (%)  Primary or Secondary (years: 7-8-9)  High school or above (above year 9) | (n = 476)  220 (46%)  256 (54%) | (n = 328)  145 (44%)  183 (56%) | *P* = 0.573 |
| Occupation  unemployed  employed  retired | (n = 480)  24 (5%)  246 (51%)  210 (44%) | (n = 331)  26 (8%)  211 (64%)  94 (28%) | ***P* < 0.001** |
| ***Acute phase Characteristics*** | | | |
| Time to initial hospital evaluation above 7 days (since onset of symptoms) | (n = 448)  316 (71%) | (n = 315)  231 (73%) | *P* = 0.398 |
| Maximal O2 consumption   1. Room air 2. Low flow 3. High flow 4. CPAP, NIMV, MV, ECMO | (n = 486)  215 (44%)  89 (18%)  108 (22%)  74 (15%) | (n = 337)  139 (41%)  80 (24%)  59 (18%)  59 (18%) | *P* = 0.107 |
| Hospital admission | 192 (39%) | 145 (43%) | *P* = 0.086 |
| More than 14 days of hospital stay  (incl. pts. with many days at Emergencies) | (n = 462)  207 (45%) | (n = 313)  135 (43%) | *P* = 0.645 |
| ICU admission | 10 (2%) | 17 (5%) | *P* = 0.017 |
| ***Follow-up evaluation*** |  |  |  |
| Median time to follow-up  (above 133 days) | 266 (55%) | 216 (64%) | *P* = 0.012 |

LEGEND: O2: oxygen; SD: standard deviation; CPAP: Continuous Positive Air pressure; MV: mechanical ventilation; ECMO: extra-corporeal membrane oxygenation; BMI: body-mass index; ICU: intensive care unit - in bold characters the significant results.

Table 3: Association between SES and acute-phase severity (O2-need)

No association was found among SES and acute-phase O2-need, as a proxy for severity, in a multiple linear regression model. SES was instead associated to male sex (linear regression, beta coefficient = 0.61; 95%CI = 0.46 to 0.77; p < 0.001). A trend to significance was observed also for age > 60 (beta coefficient = 0.22; 95%CI = 0.05 to 0.38; p = 0.010), and BMI > 30 (beta coefficient = 0.29; 95%CI = 0.10 to 0.49; p = 0.003).

|  | **Maximal O2 consumption**  **during the acute phase**  **(A to D)** | |
| --- | --- | --- |
| Adjusted R² | 0.1225 |  |
|  | *p* | beta |
| Intercept | < 0.0001 | 1.6069 |
| **Social Class (1 to 9)** | 0.3364 | -0.0168 |
| **Household members admitted for COVID** | 0.2745 | -0.1196 |
| **Number of comorbiditiees** | 0.0593 | 0.1750 |
| **Male sex** | **< 0.0001** | 0.6132 |
| **Age** | 0.0101 | 0.2151 |
| **BMI** | 0,0033 | 0.2937 |
| **Cigarette Smoking** | 0.1039 | -0.1307 |
| **History of diabetes** | 0.1382 | 0.2199 |
| **History of hypertension** | 0.9166 | -0.0142 |

REFERENCES (FOR SUPPLEMENTARY MATERIAL)

1. Galeoto G, Lauta A, Palumbo A, Castiglia SF, Mollica R, Santilli V et al. The Barthel Index: Italian Translation, Adaptation and Validation. Int J Neurol Neurother 2015 June 19, 2:2.
2. Catania G, Bell C, Ottonelli S, Marchetti M, Bryce J, Grossi A, et al. Cancer-related fatigue in Italian cancer patients: validation of the Italian version of the Brief Fatigue Inventory (BFI). Support Care Cancer. 2013 Feb;21(2):413-9. Epub 2012 Jul 13. PubMed PMID: 22790224.
3. Graham B.L., Steenbruggen I., Miller M.R., Barjaktarevic I.Z., Cooper B.G., Hall G.L., Hallstrand T.S., Kaminsky D.A., McCarthy K., McCormack M.C., Oropez C.E., Rosenfeld M., Stanojevic S., Swanney M.P., Thompson B.R.: Standardization of spirometry 2019 update. An Official American Thoracic Society and European Respiratory Society technical statement. Am. J. Respir. Crit. Care Med. 2019; 200: pp. e70-e88.
4. Pellegrino R., Viegi G., Brusasco V., Crapo R.O., Burgos F., Casaburi R., Coates A., van der Grinten C.P., Gustafsson P., Hankinson J., Jensen R., Johnson D.C., MacIntyre N., McKay R., Miller M.R., Navajas D., Pedersen O.F., Wanger J.: Interpretative strategies for lung function tests. Eur. Respir. J. 2005; 26: pp. 948-968.
5. McGowan A., Sylvester K., Burgos F., Boros P., de Jongh F., Kendrick A., Lloyd Cooper J., Kirkby J., Makonga-Braaskma J., Steenbruggen I., den Berg J.: ERS 9.1 Statement on lung function during COVID-19 Final with Contributors. Ers 2020; pp. 1-5. Accessed August 2, 2020 <https://ers.app.box.com/s/zs1uu88wy51monr0ewd990itoz4tsn2h>
6. Quanjer P.H., Stanojevic S., Cole T.J., Baur X., Hall G.L., Culver B.H., Enright P.L., Hankinson J.L., Ip M.S., Zheng J., Stocks J.: ERS Global Lung Function Initiative. Multi-ethnic reference values for spirometry for the 3-95-yr age range: the global lung function 2012 equations. Eur. Respir. J. 2012; 40: pp. 1324-1343.
7. Stanojevic S., Graham B.L., Cooper B.G., Thompson B.R., Carter K.W., Francis R.W., Hall G.L.: Global Lung Function Initiative TLCO working group; Global Lung Function Initiative (GLI) TLCO. Official ERS technical standards: Global Lung Function Initiative reference values for the carbon monoxide transfer factor for Caucasians. Eur. Respir. J. 2017; 50
8. Horowitz, M., Wilner, N., Alvarez, W., 1979. Impact of event scale: a measure of subjective stress. Psychosom. Med. 41, 209–218. https://doi.org/10.1097/ 00006842-197905000-00004.
9. Craparo, G., Faraci, P., Rotondo, G., Gori, A., 2013. The impact of event scale - revised: psychometric properties of the italian version in a sample of flood victims.Neuropsychiatr. Dis. Treat. 9, 1427–1432. <https://doi.org/10.2147/NDT.S51793>.
10. McHorney, C.A., Ware, J.E., Raczek, A.E., 1993. The MOS 36-item short-form health survey (SF-36): II. Psychometric and clinical tests of validity in measuring physical and mental health constructs. Med. Care 31, 247–263. <https://doi.org/10.1097/00005650-199303000-00006>
11. Ware, J., Sherbourne, C.D., 1992. The MOS 36-item short form health survey (SF-36): I conceptual framework and item selection. Med. Care 30, 473–483. https://doi.org/10.1097/00005650-199206000-00002.
12. Apolone, G., Mosconi, P., 1998. The italian SF-36 health survey: translation, validation and norming. J. Clin. Epidemiol. 51, 1025–1036.
